# Morphologic Analysis of Condyle among Different Disc Status in the Temporomandibular Joints by Three-dimensional Reconstructive Imaging: A Preliminary Study

**DOI:** 10.1186/s12903-022-02438-1

**Published:** 2022-09-12

**Authors:** Chen-xi Li, Xu Liu, Zhong-cheng Gong, Sakendeke Jumatai, Bin Ling

**Affiliations:** 1 Oncological Department of Oral and Maxillofacial Surgery, Xinjiang Medical University Affiliated First Hospital, No.137 Liyushan South Road, Urumqi, 830054 People’s Republic of China; 2 Department of Oral and Maxillofacial Radiology, Xinjiang Medical University Affiliated First Hospital, Urumqi, 830054 People’s Republic of China; 3 School of Stomatology, Xinjiang Medical University, Urumqi, 830011 People’s Republic of China; 4 Stomatological Research Institute of Xinjiang Uygur Autonomous Region, Urumqi, 830054 People’s Republic of China

**Keywords:** Temporomandibular joint disorders, Articular disc, Mandibular condyle, Cone beam computed tomography, Magnetic resonance imaging, Theoretical model

## Abstract

**Objectives:**

Morphological study is a common approach in the field of anterior disc displacement (ADD) pathology; however, analysis based on three-dimensional reconstructive imaging has not been investigated. This study investigated the association between ADD and the status of the mandibular condyle and articular fossa.

**Methods:**

Thirty-four patients were divided into three groups: normal articular disc position (NADP), anterior disc displacement with reduction (ADDwR), and anterior disc displacement without reduction (ADDwoR). Multiple grouped comparisons of three different disc statuses were performed by Kruskal–Wallis H test and variance analysis respectively. Receiver-operating characteristic curve was plotted to assess the diagnostic efficacy of the morphological parameters. Multivariate logistic regression analysis was used to investigate the interfering factors of ADD.

**Results:**

The condylar volume (CV) and condylar superficial area (CSA) in the NADP, ADDwR, and ADDwoR groups exhibited obvious changes (*P* < 0.05). Both CV and superior joint space (SJS) presented a good diagnostic accuracy for NADP-ADDwoR [area under the curve (AUC)_*CV*_ = 0.813; AUC_*SJS*_ = 0.855)], and ADDwR-ADDwoR (AUC_*CV*_ = 0.858; AUC_*SJS*_ = 0.801). CSA presented a good diagnostic accuracy for ADDwR-ADDwoR (AUC = 0.813). A multivariate logistic ordinal regression model showed that the CV [odds ratio (OR) = 1.011; regression coefficient (*RC*) = 0.011, *P* = 0.018], SJS (OR, 8.817; *RC* = 2.177; *P* < 0.001), and medial joint space (MJS) (OR, 1.492; *RC* = 0.400; *P* = 0.047) had a significantly impact on the groups.

**Conclusion:**

CV, CSA, SJS, and MJS were significantly associated with the different disc status, and the condyle in ADD exhibited 3-dimensionally altered dimensions. They could be considered as promising biometric markers to assess the ADD.

**Supplementary Information:**

The online version contains supplementary material available at 10.1186/s12903-022-02438-1.

## Introduction

Temporomandibular joint disorders (TMD) comprise several oral and maxillofacial diseases and frequently occur in young and middle-aged individuals. TMD affects up to 15% of adults, with a peak incidence at 20–40 years of age [1, 2]. Anterior disc displacement (ADD) occurs in people of all ages, with a high prevalence in women aged 20–40 years, resulting in clicking, joint pain, limited range of mouth opening, masticatory difficulty, mandible dysfunction, and so on [3–5].

The temporomandibular joint (TMJ) comprises soft tissue (e.g., articular disc, capsule, intracapsular ligament, extracapsular ligament) and the osseous structure; because of its complex anatomy and biomechanics, it is susceptible to pathological changes [6]. The articular disc of the TMJ is a slim, oblong plate comprising coarse nonvascular connective cells located between the condyle of the jaw and mandibular fossa. Unlike the disc itself, the retrodiscal tissue is vascular and highly innervated. Therefore, the retrodiscal tissue is often a major contributor to edema and hyperemia with ADD without reduction because of its repetitive impingement of the condyle and high tension of the stretching ligament [7], resulting in pain with speaking and eating. Furthermore, because an altered or misaligned disk–condyle structural relationship is maintained during mandibular translation, ADD without reduction can often include a history of jaw clicking with the sudden onset of the limited ability to open the mouth in the absence of clicking; therefore, its clinical characteristics include mandibular deviation to the affected side when opening the mouth and marked limited lateral movement to the opposite side [8]. When the biochemical and biomechanical loads tremendously exceed the normal levels that the temporomandibular disc can withstand, it will perforate (this occurs easily at the bilaminar zone). The cause of ADD without reduction remains unclear, but it likely comprises psychological factors, abnormal dental occlusion, immunological anomalies, and parafunctional habits [2, 9]. Several studies have examined the natural course of disc position and configuration in nonreducing ADD [10–14], showing persistent existence of disc displacement, continued disc deformity and probably accelerated bone change. Besides, Cai et al. [12] previous study showed the disc would likely become more anteriorly displaced and shortened, and reducing ADD could turn into nonreducing ADD. Clinically, ADD (with or without reduction) involves degenerative manifestations, mainly in the mandibular condyle and articular disc; furthermore, the morphological damage of these is associated with the development of ADD [15]. Magnetic resonance imaging (MRI) is the gold standard for visualizing the articular disc morphology and position. However, MRI is not useful for postprocessing image reorientation, allows for relatively few axial views, and has low spatial resolution; therefore, issues with bony components, such as the mandibular condyle, are missed by MRI [16]. Several studies have found that there is a certain relationship between the condylar dimension and ADD; however, most results of those studies only apply to MRI assessments [17, 18]. Additionally, it is well-known that cone beam computed tomography (CBCT) is superior to multidetector computed tomography (MDCT) for scanning and imaging the bony structures of the TMJ. CBCT provides clearer images, more images with higher quality, requires shorter acquisition time, involves a lower radiation dose, and is less expensive [19].

The purpose of this imaging study was to quantitatively evaluate changes in the morphology and inclination of the mandibular condyle among different position of the articular disc, as well as analyzing the influential factors of changes in disc status, to achieve more insights into the natural course of ADD of the TMJ. The hypothesis proposed herein refers to that the alterations of mandibular condyle may dictate the risk level for disc displacement.

## Patients and methods

### Study design

To address the research purpose, the investigators designed and implemented a cross-sectional retrospective study. The study population was composed of all patients presenting for evaluation and management of TMJ ADD recruiting from the Temporomandibular Joint Specialist Clinic, The First Affiliated Hospital of Xinjiang Medical University, China, between March 2018 and May 2021. The study protocol was approved by the Ethics Committee of the Stomatological School of Xinjiang Medical University, The First Affiliated Hospital of Xinjiang Medical University (approval no. K202108-25) and followed the principles outlined in the Declaration of Helsinki. Informed consent was provided by all families. All data generated or analyzed during this study are included in this published article.

According to the *Research Diagnostic Criteria for Temporomandibular Disorders* (RDC/TMD) [20], the diagnostic key points of ADD include the following: TMJ clicking sounds or noises during different time phases of opening and closing movements; various degrees of limitation in opening the mouth; mandible deflection when opening the mouth; and pain confined to the TMJ area at rest and during function.

### Study sample

#### Inclusion criteria

The inclusion criteria were as follows: ADD with or without reduction that had not been treated previously; no history of infection, trauma, and tumor in the otic area or TMJ; ability to undergo plain and enhanced MRI examinations; high-resolution CBCT scan of the TMJ within 3 months of the MRI examination; and willingness to accept our medical treatment.

#### Exclusion criteria

Patients who met any of the following criteria were excluded: obvious organ dysfunction or organ failure; radiographic examination showed organic lesions in the TMJ; a history of osteoarthritis involved with the TMJ (e.g., juvenile idiopathic arthritis); congenital cranio-maxillo-facial anomalies (e.g., condylar hypertrophy) and/or any other TMJ disease; and contraindications for MRI and CBCT examinations.

#### MR image acquisition

A 3-Tesla system equipped with the multichannel transcranial magnetic stimulation/MRI head-neck coil array (MAGNETOM Aera; Siemens Healthineers, Erlangen, Germany) was used during the resting-state functional MRI examination of the bilateral TMJ without sedatives or intravenous contrast medium. The patient was kept in the supine position so that the Frankfurt horizontal plane was perpendicular to the table surface. Scanning was performed in the oblique sagittal (wide open and closed mouth), axial (wide open and closed mouth), and coronal (closed mouth) planes so that the projection angle was in line with Schüller’s position. Fast spin echo sequences using T1-weighted imaging, T2-weighted imaging, and proton density-weighted imaging generating contiguous sections of 20 axial slices, 15 coronal slices, and 18 sagittal slices, respectively, were used to evaluate different TMJ segments. The technical parameters for T1-weighted imaging, T2-weighted imaging, and proton density-weighted imaging, respectively, were as follows: repetition time (TR) = 700 ms, echo time (TE) = 10 ms, flip angle = 120°, field of view (FOV) = 25.6 cm × 25.6 cm, matrix = 256 × 256, number of acquisitions = 1, slice thickness = 2 mm, and slice gap = 0.2 mm; TR/TE = 5000 ms/92.5 ms, flip angle = 120°, FOV = 21 cm × 21 cm, matrix = 320 × 288, number of acquisitions = 2, slice thickness = 3 mm, and slice gap = 4 mm; and TR/TE = 3000 ms/64 ms, flip angle = 120°, FOV = 14 cm × 14 cm, matrix = 288 × 192, number of acquisitions = 2, slice thickness = 2 mm, and slice gap = 1 mm. All imaging protocols were identical for all patients. All magnetic resonance images were analyzed by two clinicians (a radiologist and an oral and maxillofacial specialist).

#### CBCT image acquisition

All patients underwent high-resolution CBCT of the TMJ under uniform conditions. A head positioner and cursor positioning system were used to position the midsagittal plane of the face of the patient vertical to the ground and the Frankfurt horizontal plane parallel to the ground. Patients remained immobile in the mandibular postural position (binocular smooth inspect facing forward, no chewing, no swallowing, no speaking, and the upper and lower dentitions naturally maintaining the intercuspal position) during the scanning procedure. The technical parameters were as follows: tube voltage, 85 kilovolt peak; effective tube current, 7 mA; thickness layer of the scanning process, 0.15 mm; reconstructed slice thickness, 0.625 mm; reconstructive interval, 0.5 mm; revolution speed, 1 s/rotation; and matrix, 512 × 512. The CBCT protocol included the GALILEOS® COMFORTPLUS (Sirona Dental Systems GmbH, Bensheim, Germany) unit with a FOV of 20 × 19 cm, isotropic voxels of 0.3 mm in the axial slice thickness, and 15 s of total scanning time (Additional file 1).

#### Processing of imaging materials and data measurements

**Fig. 1:**
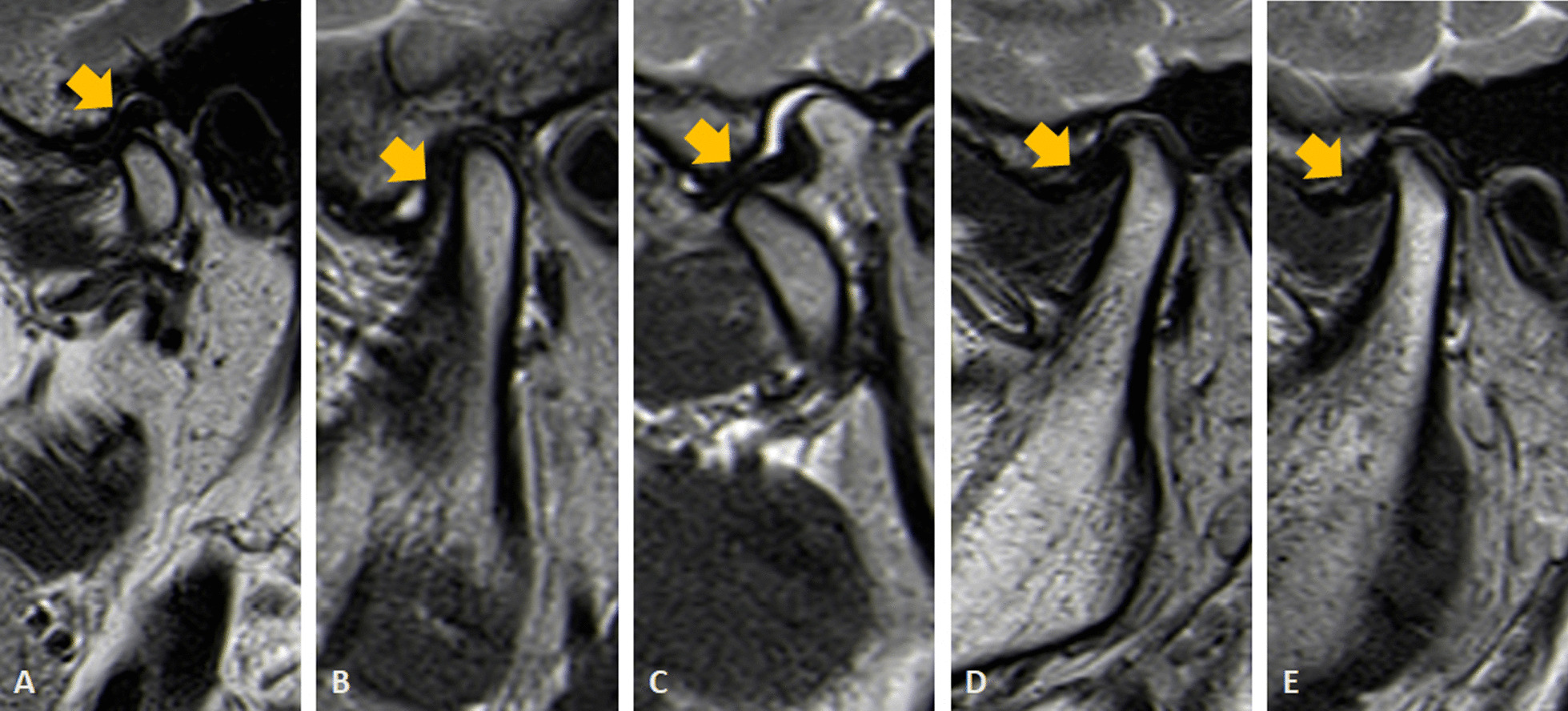
Different functional types of anterior disc displacement of the temporomandibular joint (TMJ) according to oblique sagittal proton density-weighted imaging (PDWI) (indicated by arrows). **A**. Normal articular disc position (NADP). **B** and **C**. Anterior disc displacement with reduction (ADDwR). **D** and **E**. Anterior disc displacement without reduction (ADDwoR). **B** and** D**. Closed mouth position. **C** and** E**. Wide open position

A picture archiving and communication system workstation created the TMJ MRI data. All MRI performed using the DICOM format were processed using ImageJ software version 1.52 (National Institutes of Health, Bethesda, Maryland, USA) to classify the following three subtypes based on the TMJ disc location [21]: normal articular disc position (NADP); ADD with reduction (ADDwR); and ADD without reduction (ADDwoR) (Fig. 1A–E).

**Fig. 2:**
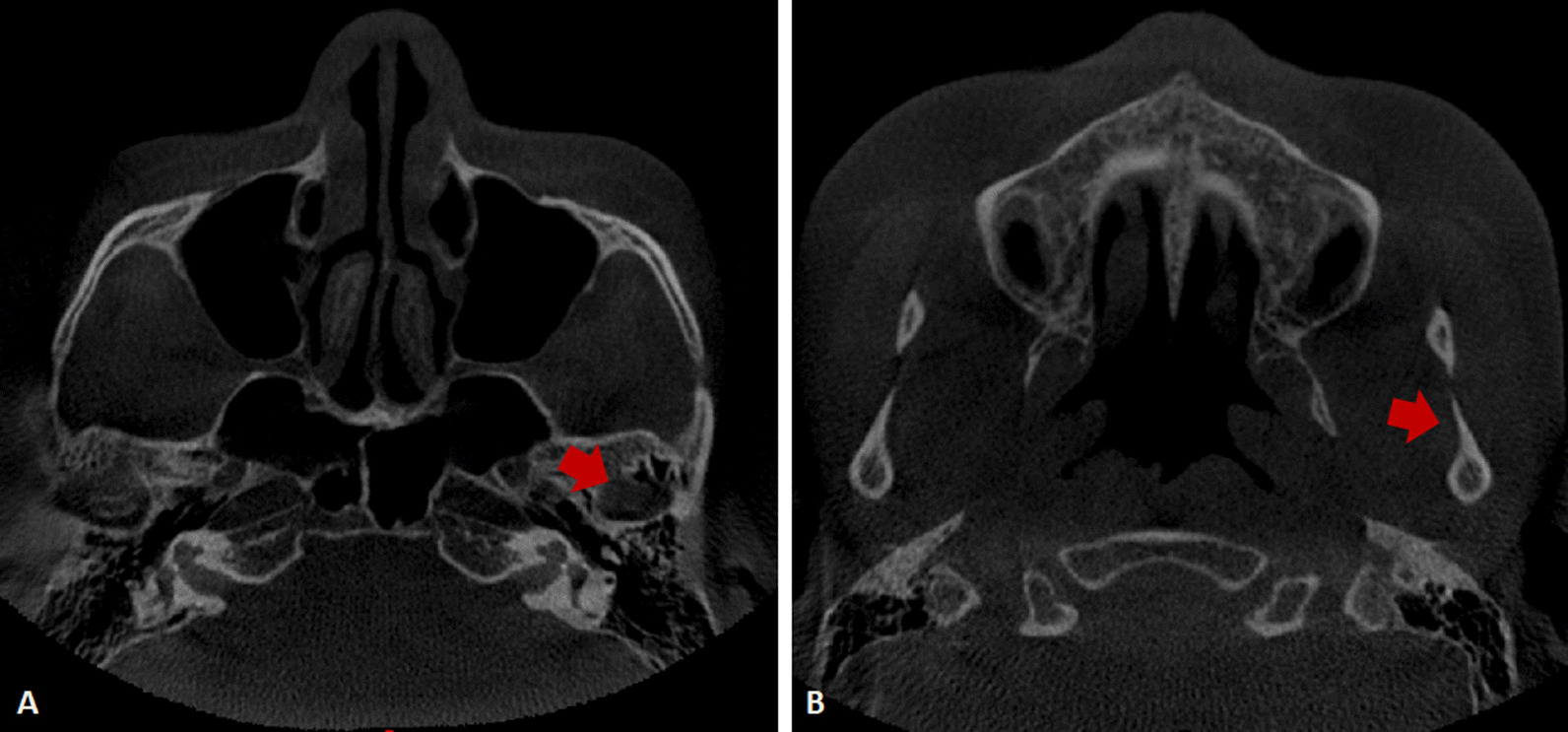
Determination of the condylar boundary in the coronal plane. **A**. Appearance of the first high-density shadow defined as the top of the condyle. **B** First separation of the coracoid process and condyle is regarded as the bottom of the condyle

**Fig. 3:**
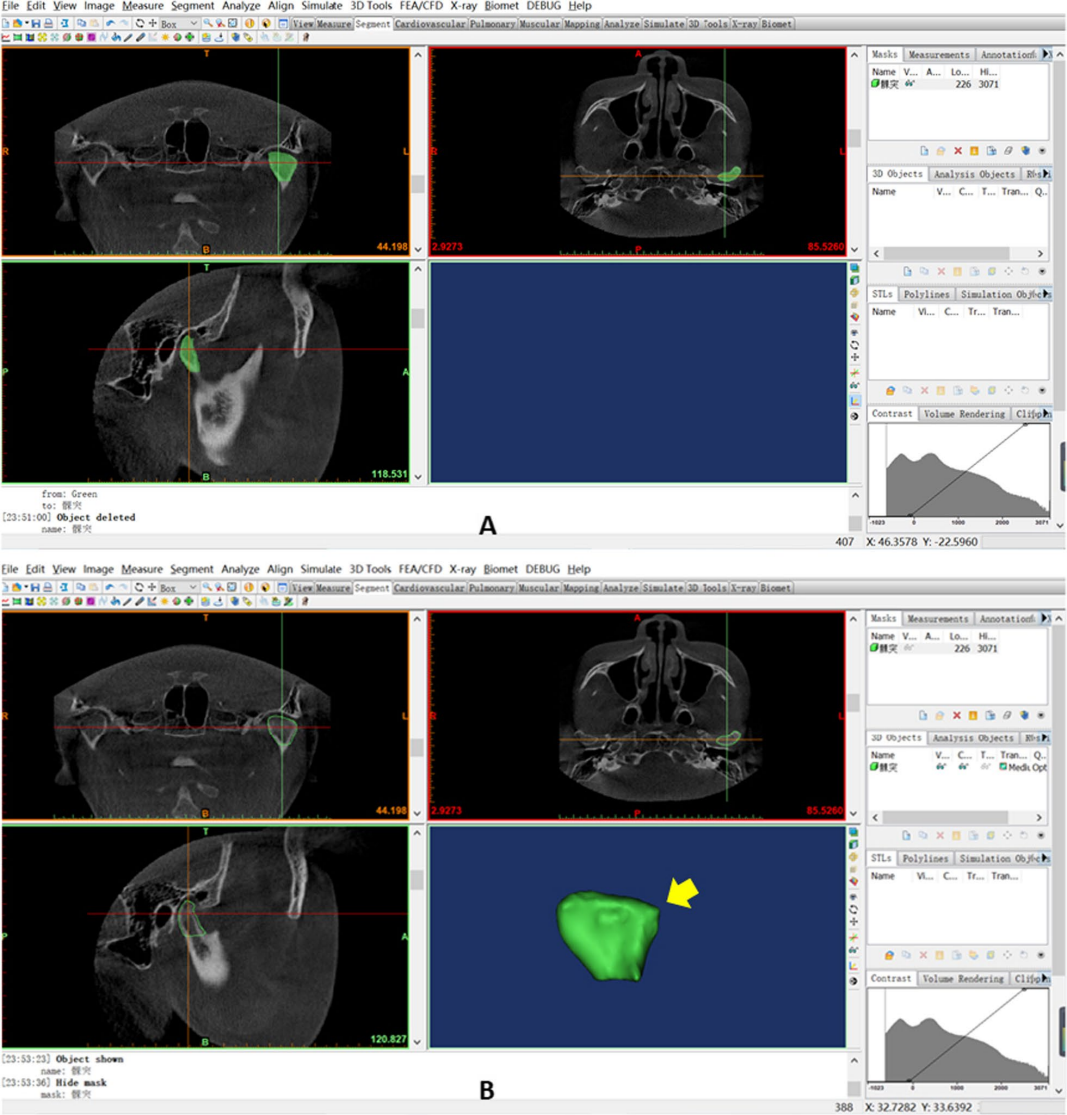
Reconstruction of the condyloid process. **A**. Use of the Multiple Slice Edit plugin to select the condylar range within its boundary at the coronal, axial, and sagittal levels. **B**. Using Smooth and Wrap instruction to refine the contour. The three-dimensional (3D) reconstructed condyle is modeled (indicated by a yellow arrow)

**Fig. 4:**
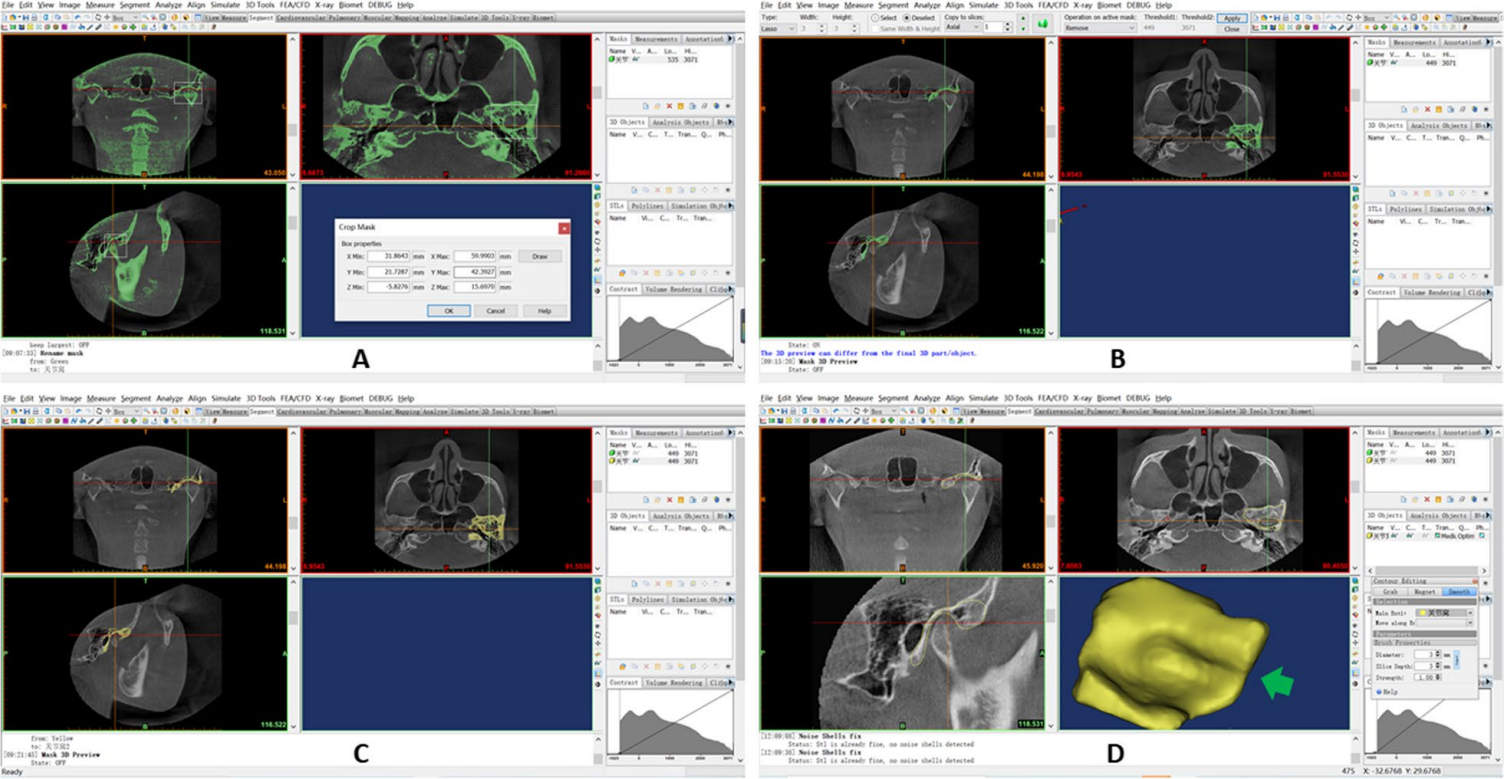
Reconstruction of the articular fossa. **A**. Use of the Crop Mask command to select the region of articular fossa. **B**. Use of the Multiple Slice Edit plugin to erase the partial condyle at the coronal, axial, and sagittal levels. **C**. Further removal of the condyle via the Region Growing command; **D**. Use of the Smooth and Wrap command to refine the contour. The three-dimensional (3D) reconstructed fossa is modeled (pointed by a green arrow)

CBCT images were exported to the SIDEXIS XG Digital Radiography system (Sirona Dental Systems GmbH, Bensheim, Germany) and imported to Mimics software version 19.0 (Materialise Inc., Leuven, Belgium) for 3D plane reorientation and reconstruction. The 3D parameterized modeling was performed by reorienting every plane, setting the grayscale thresholds (226–3071 HU), determining the condyle boundary (Fig. 2A, B), completing 3D reconstruction of the condyle (Fig. 3A, B), and completing 3D reconstruction of the glenoid fossa (Fig. 4A–D).

**Fig. 5:**
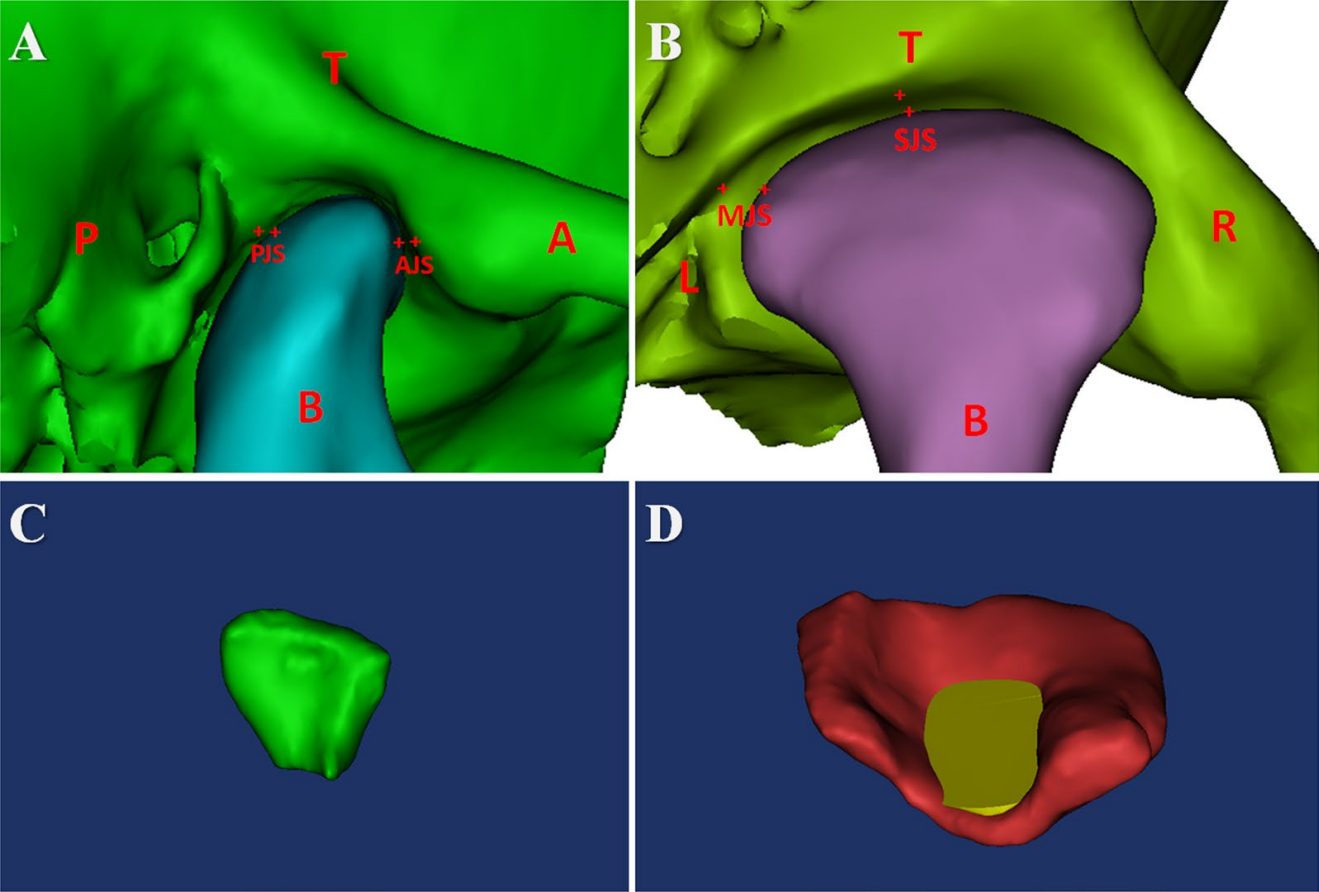
Measurements of parameters on the reconstructive imaging of 3D model. In **A** and **B**: 3D = three-dimensional; A = the anterior direction; AJS = anterior joint space; B = the bottom direction; L = the left direction; MJS = medial joint space; P = the posterior direction; PJS = posterior joint space; R = the right direction; SJS = superior joint space; T = the top direction In **C** and **D**: Reconstructed condyle was indicated as green color; Reconstructed glenoid fossa was indicated as red color; The portion of condyle in the glenoid fossa was indicated as yellow color

Using the reconstructed 3D model, 3-matic Research software version 11.0 (Materialise Inc., Leuven, Belgium), Geomagic Wrap 2017 (64bit) (Raindrop 3D Systems Inc., Wilmington, North Carolina, USA), and Mimics software version 19.0 (Materialise Inc., Leuven, Belgium), the following 10 representative morphological parameters were calculated and output automatically through the above measuring softwares: condylar volume (CV); condylar superficial area (CSA); fossa volume (FV); fossa superficial area (FSA); the proportion of the condylar volume in the articular fossa (CV%); the proportion of the condylar superficial area in the articular fossa (CSA%). The definition of measured space is based on the spatial relationship between the condyle and the articular fossa. a. anterior joint space (AJS), the shortest distance between the front of condyle and the front of fossa; b. medial joint space (MJS), the shortest distance between the medial side of condyle and the medial side of fossa; c. posterior joint space (PJS), the shortest distance between the posterior side of condyle and the posterior side of articular fossa; d. superior joint space (SJS), the shortest distance from the uppermost point of the condyle to the articular fossa (Fig. 5A, B). Based on the reconstruction of condyle and fossa, the CV, CSA, FV, and FSA values were calculated and output automatically when double-clicked on the icon in display interface (Fig. 5C, D); and, correspondingly, the CV% value was evaluated using the following formula: CV% =|CV_*condyle*_-CV_*fossa*_|÷ CV_*condyle*_; the CSA% value was evaluated using the following formula: CSA% =|CSA_*condyle*_-CSA_*fossa*_|÷ CSA_*condyle*_.

### Statistical analysis

Statistical analysis was performed using Statistical Package for Natural Science (version 26.0; IBM SPSS; Armonk, New York, USA). The Kolmogorov–Smirnov test was used to verify the normality of all data. Normally distributed data are expressed as mean ± standard deviation (SD) ($\overline{x} \pm s$). Non-normally distributed data are presented as quartile (25^th^ percentile, 75^th^ percentile). Pairwise methods were used to perform multiple comparisons. The Kruskal–Wallis H test was performed for the non-normal data. A one-way analysis of variance followed by a least significant difference post hoc analysis was performed for normal data with homoscedasticity. Dunnett’s T3 post hoc test was performed for normal data with heteroscedasticity. *P* < 0.05 was considered statistically significant. GraphPad Prism software version 6.0 (Graph Pad Software Inc., San Diego, California, USA) was used to plot values. The receiver-operating characteristic (ROC) curve was analyzed to assess the diagnostic efficacy of morphological parameters. By calculating the area under the curve (AUC), the diagnostic accuracy was graded as follows: excellent, 0.9–1.0; good, 0.8–0.9; fair, 0.7–0.8; poor, 0.6–0.7; and failure, 0.5–0.6 [22]. A multivariate logistic ordinal regression analysis was used to investigate factors associated with TMJ ADD. A predictive model was generated based on the results of the multivariate logistic ordinal regression analysis. R software version 4.0.4 (R Foundation for Statistical Computing, Vienna, Austria) was used for mathematical model construction.

## Results

A total of 34 patients (68 joints) met the inclusion criteria. All the patients were affected unilaterally, in detail, 17 patients ADD with reduction, and 17 patients ADD without reduction. Therefore, a self-control grouping (affected and healthy side of TMJ) was determined. Patient age ranged from 16 to 51 years (average, 29.10 ± 8.94 years), and the female-to-male ratio was 5.8:1 (29 females, 5 males). For the duration of disease, most cases of TMJ ADD lasted more than 4 months (*N*_*(ADDwR)*_ = *N*_*(ADDwoR)*_ = 13). In regards to the occlusal interference, locked bite was the predominant factor (*N*_*(ADDwR)*_ = 4) for the ADD with reduction; the majority was third-molar elongation (*N*_*(ADDwoR)*_ = 5), followed by open bite (*N*_*(ADDwoR)*_ = 3) in the ADD without reduction (Table 1).

**Table 1:** Clinical baselined information of patients

Parameters	Classification	ADDwR (*N*)	ADDwoR (*N*)
*Gender*			
	Male	2	3
	Female	15	14
*Age (years)*			
	≤ 20	2	6
	20 ~ 40	12	11
	≥ 40	3	0
*Duration of disease (months)*			
	< 4	4	4
	≥ 4	13	13
*Occlusal interference*			
	No obvious malocclusion	1	1
	Edge-to-edge bite	2	2
	Deep overbite	2	2
	Deep overjet	1	2
	Crossbite	2	1
	Open bite	2	3
	Locked bite	4	1
	Third-molar elongation	3	5
Total number	34	17	17

*ADDwR* Anterior disc displacement with reduction, *ADDwoR* Anterior disc displacement without reduction

The Kolmogorov–Smirnov test result showed that the CSA, FV, FSA, CV%, CSA%, and MJS conformed to the Gaussian distribution (Sig > 0.05); however, the CV, SJS, AJS, and PJS data were non-normally distributed. The results of the pairwise comparisons among three different disc displacement types indicated that the CV was significantly lower in the ADD without reduction group than in the NADP (*P* = 0.001) and ADD with reduction groups (*P* = 0.001). The CSA was also significantly in the ADD without reduction group than in the NADP (*P* < 0.001) and ADD with reduction groups (*P* = 0.004). The CV% and CSA% were statistically significant in the NADP (CV%: 22.435 ± 13.247; CSA%: 7.194 ± 5.798) and ADD without reduction (CV% 30.165 ± 12.210; CSA%: 11.506 ± 5.827) groups (CV%: *F* = 1.285 and *P*_*(NADP-ADDwoR)*_ = 0.016; CSA%: *F* = 3.066 and *P*_*(NADP-ADDwoR)*_ = 0.028). The SJS of the NADP group (25^th^ percentile, 1.435 mm; 75^th^ percentile, 2.695 mm) was larger than that of the ADD without reduction group (25^th^ percentile, 0.860 mm; 75^th^ percentile, 1.315 mm). The SJS of the ADD with reduction group (25^th^ percentile, 1.235 mm; 75^th^ percentile, 2.345 mm) was also larger than that of the ADD without reduction group (*H* = 15.141; *P*_*(NADP-ADDwoR)*_ < 0.001; *P*_*(ADDwR-ADDwoR)*_ = 0.005). The MJS was statistically significantly different between the NADP group (5.072 ± 1.574 mm) and ADD with reduction group (3.682 ± 1.454 mm), as well as between the ADD with reduction group and ADD without reduction group (5.075 ± 1.761 mm) (*F* = 4.281; *P*_*(NADP-ADDwR)*_ = *P*_*(ADDwR-ADDwoR)*_ = 0.015) (Table 2).

**Table 2:** Comparisons of morphological parameters of TMJ according to different disc displacement types

Variables	NADP	ADDwR	ADDwoR	*P*_*K-S*_ value	*H* or* F* value	Significance
CV (mm^3^)	1566.775 2100.115	1424.365 2066.185	919.505 1642.935	0.029	15.046	*P*_*(NADP-ADDwR)*_ = 0.890 *P*_*(NADP-ADDwoR)*_ = 0.001 *P*_*(ADDwR-ADDwoR)*_ = 0.001
CSA (mm^2^)	886.312 ± 206.735	846.356 ± 104.682	689.275 ± 124.039	0.147	8.009	*P*_*(NADP-ADDwR)*_ = 0.446 *P*_*(NADP-ADDwoR)*_ < 0.001 *P*_*(ADDwR-ADDwoR)*_ = 0.004
FV (mm^3^)	652.068 ± 318.949	580.804 ± 193.351	642.784 ± 248.556	0.200	0.381	*P*_*(NADP-ADDwR)*_ = 0.426 *P*_*(NADP-ADDwoR)*_ = 0.917 *P*_*(ADDwR-ADDwoR)*_ = 0.488
FSA (mm^2^)	583.897 ± 148.062	510.611 ± 101.293	543.922 ± 117.772	0.200	0.357	*P*_*(NADP-ADDwR)*_ = 0.509 *P*_*(NADP-ADDwoR)*_ = 0.906 *P*_*(ADDwR-ADDwoR)*_ = 0.437
CV%	22.435 ± 13.247	25.788 ± 16.479	30.165 ± 12.210	0.200	1.285	*P*_*(NADP-ADDwR)*_ = 0.491 *P*_*(NADP-ADDwoR)*_ = 0.016 *P*_*(ADDwR-ADDwoR)*_ = 0.370
CSA%	7.194 ± 5.798	7.729 ± 4.928	11.506 ± 5.827	0.200	3.066	*P*_*(NADP-ADDwR)*_ = 0.779 *P*_*(NADP-ADDwoR)*_ = 0.028 *P*_*(ADDwR-ADDwoR)*_ = 0.052
SJS (mm)	1.435 2.695	1.235 2.345	0.860 1.315	0.008	15.141	*P*_*(NADP-ADDwR)*_ = 0.371 *P*_*(NADP-ADDwoR)*_ < 0.001 *P*_*(ADDwR-ADDwoR)*_ = 0.005
AJS (mm)	0.820 1.765	1.190 2.850	0.950 2.530	0.006	1.368	*P* = 0.505
PJS (mm)	1.725 2.835	1.305 2.605	1.545 2.995	0.000	1.545	*P* = 0.462
MJS (mm)	5.072 ± 1.574	3.682 ± 1.454	5.075 ± 1.761	0.200	4.281	*P*_*(NADP-ADDwR)*_ = 0.015 *P*_*(NADP-ADDwoR)*_ = 0.997 *P*_*(ADDwR-ADDwoR)*_ = 0.015

*ADDwR* Anterior disc displacement with reduction, *ADDwoR* Anterior disc displacement without reduction, *CV* Condylar volume, *CV%* The proportion of condylar volume in the articular fossa, *CSA* Condylar superficial area, *CSA%* The proportion of the condylar superficial area in the articular fossa, *FV* Fossa volume, *FSA* Fossa superficial area, *AJS* Anterior joint space, *K-S* Kolmogorov–Smirnov, *MJS* Medial joint space, *NADP* Normal articular disc position, *PJS* Posterior joint space, *SJS* Superior joint space

Both the CV and SJS showed good diagnostic accuracy for the NADP and ADD without reduction groups (AUC_*CV*_ = 0.813; AUC_*SJS*_ = 0.855), and for the ADD with reduction and ADD without reduction groups (AUC_*CV*_ = 0.858; AUC_*SJS*_ = 0.801). The CSA showed good diagnostic accuracy for the ADD with reduction and ADD without reduction groups (AUC = 0.813). The CSA showed fair diagnostic accuracy for the NADP and ADD without reduction groups (AUC = 0.789). The MJS showed fair accuracy for the NADP and ADD with reduction groups (AUC = 0.751), and for the ADD with reduction and ADD without reduction groups (AUC = 0.723) (Table 3).

**Table 3:** ROC analysis of the CV, CSA, SJS, and MJS among NADP, ADDwR, and ADDwoR

Significant parameters	AUC	95% CI	Cut-off value	Sensitivity (%)	Specificity (%)
*CV*					
NADP to ADDwoR	0.813	0.301–0.703	1676.485	64.7	94.1
ADDwR to ADDwoR	0.858	0.733–0.983	1683.210	70.6	94.1
*CSA*					
NADP to ADDwoR	0.789	0.636–0.941	725.44	82.4	70.6
ADDwR to ADDwoR	0.813	0.664–0.962	736.51	88.2	70.6
*SJS*					
NADP to ADDwoR	0.855	0.728–0.982	1.355	82.4	82.4
ADDwR to ADDwoR	0.801	0.654–0.948	1.205	88.2	58.8
*MJS*					
NADP to ADDwR	0.751	0.584–0.917	3.995	82.4	64.7
ADDwR to ADDwoR	0.723	0.546–0.900	2.580	82.4	52.6

*ADDwR* Anterior disc displacement with reduction, *ADDwoR* Anterior disc displacement without reduction, *AUC* Area under curve, *CI* Confidence interval, *CV* Condylar volume, *CSA* Condylar superficial area, *MJS* Medial joint space, *NADP* Normal articular disc position, *ROC* Receiver-operating characteristic, *SJS* Superior joint space

The multivariate logistic ordinal regression analysis showed that the CV (odds ratio [OR], 1.011), CSA (OR, 0.976), SJS (OR, 8.817), and MJS (OR, 1.492) were risk factors in the ADD without reduction groups. Among them, the CV (regression Coefficient [RC] = 0.011; *P* = 0.018), SJS (RC = 2.177; *P* < 0.001) and MJS (RC = 0.400; *P* = 0.047) had a significantly positive impact on the groups, whereas the CSA (RC = -0.024, *P* = 0.076) did not impact the groups (Table 4). Based on the results of the multivariate logistic ordinal regression analysis combined with logit probit, the CV, CSA, SJS, and MJS could be used to construct a mathematical model to predict ADD without reduction. The likelihood ratio test indicated that our model efficiently predicted TMJ ADD (*χ*^*2*^ = 35.879; *P* < 0.001) (Table 5) using the following equations: logit[P(Group ≤ 0.0)/(1-P(Group ≤ 0.0))] = 2.886 + 0.011*CV-0.024*CSA + 2.177*SJS + 0.400*MJS and logit[P(Group <  = 1.0)/(1-P(Group <  = 1.0))] = 5.300 + 0.011*CV-0.024*CSA + 2.177*SJS + 0.400*MJS. The overall predictive accuracy of the model was 66.67%, indicating an acceptable fit (Table 6).

**Table 4:** Multivariate logistic ordinal regression analysis of the CV, CSA, SJS, and MJS among NADP, ADDwR, and ADDwoR

Parameters	Item	RC value	SE	*Z* value	Wald *χ*^*2*^	*P* value	OR value	95% CI
Dependent variable threshold	0.0	2.886	3.514	0.821	0.675	0.411	0.056	0.000 ~ 54.679
	1.0	5.300	3.608	1.469	2.158	0.142	0.005	0.000 ~ 5.881
Independent variable	CV	0.011	0.005	2.371	5.620	0.018	1.011	1.002 ~ 1.020
	CSA	-0.024	0.014	-1.775	3.152	0.076	0.976	0.950 ~ 1.003
	SJS	2.177	0.603	3.607	13.011	0.000	8.817	2.702 ~ 28.772
	MJS	0.400	0.202	1.982	3.928	0.047	1.492	1.004 ~ 2.216

*ADDwR* Anterior disc displacement with reduction, *ADDwoR* Anterior disc displacement without reduction, *CI* Confidence interval, *CV* Condylar volume, *CSA* Condylar superficial area, *MJS* Medial joint space, *NADP* Normal articular disc position, *OR* Odds ratio, *RC* Regression coefficient, *SE* Standard error, *SJS* Superior joint space

McFadden *R*^2^ = 0.420

Cox and Snell *R*^2^ = 0.505

Nagelkerke *R*^2^ = 0.569

**Table 5:** Likelihood ratio test of the predictive model of multivariate logistic ordinal regression

Model	-2 Logarithmic likelihood value	*χ*^*2*^ value	df	*P* value	AIC value	BIC value
Null model	112.058					
Final model	76.161	35.897	4	0.000	88.161	99.752

*AIC* Akaike information criterion, *BIC* Bayesian information criterion, *df* degrees of freedom

**Table 6:** Predictive accuracy of the multivariate logistic ordinal regression model

Item	Actual frequency	Predictive frequency	Predictive accuracy (%)
0.0	17	15	88.235
1.0	17	9	52.941
2.0	17	10	58.824
Total	51	34	66.667

## Discussion

As a crucial aspect of biomechanical research, morphological analyses have been widely used in clinical research because their intuitive understanding of the structural characteristics and simple method of obtaining data. The morphological study of TMJ has important application value in the field of TMD pathology [23]; however, most morphological studies and clinical diagnoses have been based on two-dimensional (2D) plane images. Some studies have explored evidence indicating that 3D measurements are better able to ensure the precision of the biometric results than 2D measurement. By comparing and analyzing the morphological parameters of the TMJ of asymptomatic patients using 2D and 3D measuring methods, it was observed that 2D examinations may be biased because of inaccurate positioning, which could lead to incorrect clinical diagnoses [24, 25]. Previous studies have been limited to 2D evaluations of the TMJ [18, 26–28] or have studied only the changes in the condylar position after sagittal split ramus osteotomy in the 3D space [29, 30]. Some clinical observations have indicated a close relationship between condylar changes (e.g., distal inclination, decreased height) and TMJ ADD [31, 32]. Regrettably, it has not been definitely established which dimension of the condyle is influenced by ADD because most investigations used 2D images or MRI for analyses [18, 28]. However, it has been determined that the morphological changes are caused by regressive alterations in the condyles that are associated with ADD [33]. Furthermore, a few studies preliminarily explored the 3D morphological variations of the condylar dimensions using CBCT images; nevertheless, they did not define the status of TMJ ADD using MRI [16, 34]. The present study is the first to explore the changes in the dimensions of the condyle and glenoid fossa with regard to the TMJ ADD status by applying 3D stereoscopic models reconstructed from CBCT images. The results of the present study indicated that condyles with ADD are quantitatively different from condyles with NADP.

Before performing the biometric process, we examined and distinguished the resting-state functional MR images of the temporomandibular disc position to create precise subgroups. Our study showed that both the CV and CSA dimensions diminished from NADP to ADD without reduction, which explained that the abnormal position of the temporomandibular disc was in line with the degenerative alteration of the condyle. Insufficient CV can lead to anomalies of the relative position between the condyle and articular disc, which will lead to TMJ ADD and is consistent with the clinical symptoms of TMJ internal derangement. Additionally, the condylar alteration could cause ADD and other degenerative TMJ diseases [27, 35, 36], indirectly indicating that ADD with reduction would deteriorate to ADD without reduction if appropriate treatment was not administered, leading more serious symptoms such as articular disc perforation. This decreasing trend of CV and CSA were reflected in the ADD with reduction and ADD without reduction groups. The continually decreasing condyle would also change the stress direction/trajectory of the condyle, affect the stress distribution of the articular disc, and further aggravate TMD. Nebbe et al. [37] reported that ADD with reduction might accelerate disc deformity and condylar degeneration, resulting in mandibular dysmorphosis.

The available scientific literature regarding measuring the glenoid fossa is nonexistent. However, we were able to accurately determine the fossa-related parameters. In contrast to the CV and CSA, the FV and FSA were lower in the ADD with reduction group than in the NADP group but higher in the ADD without reduction group. We considered that fossa attrition occurred with ADD with reduction because of the anomalous dynamic relationship among the condyle, disc, and fossa; however, the fossa might experience progressively compensatory growth during the transition from ADD with reduction to ADD without reduction. Although this was not statistically significant, this “irregularity” was still embodied by the objective values. The CV% and CSA% both increased gradually during the transformation from NADP to ADD with reduction to ADD without reduction, which explained how the displaced disc pushed the condyle to the articular fossa. Furthermore, during the early stage of the lesion, the temporomandibular disc is not completely deformed, and its location is in front of the condyloid process, pushing on the condyloid process with more force. The more obvious the pushing effect, the greater the proportion of condyle in the fossa. Correspondently, temporomandibular disc displacement involves the limited ability to open the mouth with ADD with reduction and with ADD without reduction in clinical practice.

The variations of all linear dimensions aligned closely with the TMJ ADD status. With progression from NADP to ADD with reduction to ADD without reduction, the displaced articular disc continuously pushes the condyloid process to the glenoid fossa, so the SJS is reduced incrementally. Furthermore, with the biofunction of the lateral pterygoid muscle, it is capable of pulling the articular disc inward and forward; therefore, the MJS with ADD with reduction was smaller than that with NADP. However, when the disc was irreversibly distorted, it could rotate medially, resulting in an increased MJS with ADD without reduction, which could be discovered by MRI. Because the mouth-opening status changes with progression from NADP to ADD with reduction to ADD without reduction, the disc should be placed in front of the condyle, resulting in limited mouth-opening ability, so the AJS is widened and the PJS is narrowed; nevertheless, when the limitation disappears during the later stage, the disc should be located in the neck of the condyle to create changes in the AJS and PJS.

The ROC analysis demonstrated that the CV and SJS could detect the morphological changes of the condyloid process during the progression from ADD with reduction and ADD without reduction and distinguish ADD from NADP. Additionally, the CSA can precisely assess the condyle size occurring with ADD with reduction, thereby distinguishing ADD with reduction from ADD without reduction. Therefore, the CV, CSA and SJS could be considered effective and persuasive biometric markers when evaluating the osseous structure of the TMJ in patients with ADD without reduction. Most importantly, a mathematical model was generated for the first time for TMJ ADD prediction. The CV, SJS, and MJS had a significantly positive influence on the groups. Further calibration curves and nomograms could be applied to assess the agreement of the nomogram-predicted probability with the actual observed probability during future multicenter studies including a larger sample size.

Because our sample size was small, our findings should be verified by studies involving larger sample sizes and other morphological parameters. Furthermore, another limitation of the present investigation include the restricted population in the 3 groups, lack of sex and age matching, the potential association between these parameters of ADD and different age and sex distributions should be clarified. In addition, the sample size of men was smaller than that of women, reflecting the lower prevalence of TMJ ADD in men [37]. Longitudinal studies with sufficient male samples are needed to better understand the relationship between condylar volume and TMJ ADD. Although cross-sectional retrospective studies are important, compared to randomized clinical trials, they have less scientific evidences. The observational design limits the degree to which cause and effect relationships can be inferred from the associations observed; a longitudinal study would be more appropriate to assert a cause-effect relationship. Secondary research containing systematic reviews and meta-analyses are needed to further confirm our findings based on a reliable sample size in the future.

## Conclusion

During this study, for the first time, we analyzed the effects of TMJ ADD on the biometric dimensions of the osseous structure by applying 3D stereoscopic models reconstructed based on the combination of MRI and CBCT. The CV, CSA, SJS, and MJS may be associated with the different disc displacement types, and the condyle in TMJ ADD exhibited altered dimensions. Therefore, they could be considered promising biometric markers for assessing the ADD status. This nomogram prediction model will help enable early diagnosis of TMJ ADD.

## Supplementary Information

**Additional file 1:** **Table S1.** Conditions that may mimic temporomandibular disc displacement without reduction.

## Data Availability

The data that support the findings of this study are available from the First Affiliated Hospital of Xinjiang Medical University (the Stomatological Hospital of Xinjiang Medical University), but restrictions apply to the availability of these data, which were used under license for the current study, and so are not publicly available. Data are however available from the authors upon reasonable request and with permission of the authors’ institute.

## Abbreviations

ADD: Anterior disc displacement
ADDwR: Anterior disc displacement with reduction
ADDwoR: Anterior disc displacement without reduction
AJS: Anterior joint space
CBCT: Cone beam computed tomography
CSA: Condylar superficial area
CSA%: The proportion of the condylar superficial area in the articular fossa
CV: Condylar volume
CV%: The proportion of condylar volume in the articular fossa
FV: Fossa volume
FSA: Fossa superficial area
K-S: Kolmogorov–Smirnov
MJS: Medial joint space
MRI: Magnetic resonance imaging
NADP: Normal articular disc position
PJS: Posterior joint space
SJS: Superior joint space
TMJ: Temporomandibular joint
