# Supplementary material for: Morphologic Analysis of Condyle among Different Disc Status in the Temporomandibular Joints by Three-dimensional Reconstructive Imaging: A Preliminary Study
Source: BMC Oral Health. 2022 Sep 12;22:395. doi: 10.1186/s12903-022-02438-1 (PMC9465965; doi:10.1186/s12903-022-02438-1)
Supplement: Supplementary file 1 — Additional file 1: Table S1. Conditions that may mimic temporomandibular disc displacement without reduction. [file 12903_2022_2438_MOESM1_ESM.docx]

**Additional file 1: Table S1. Conditions that may mimic temporomandibular disc displacement without reduction**

| **Disorder** | **Location** | **Characteristics** | **Aggravating/associated factors** | **Typical findings** | **Special examination** | **Management** |
| --- | --- | --- | --- | --- | --- | --- |
| Pyogenic arthritis | With arthralgia, the pain is often more localized around the joint | Local or systemic suppurative lesions (sometimes cannot be found) | Hematogenous infection in the whole body, (open) joint injury | Wound, bacteria in blood circulation, and synovial fluid | Increased body temperature is concurrent with increased of inflammatory cytokines | Antibiotics, irrigation |
| Osteoarthritis | With arthralgia, the pain is often more localized around the joint | Stiffness, tenderness, swelling, pain or aching in more than one joint | HLA class II genotypes, smoking, and obesity can make arthritis worse | The same symptoms on both sides of the body | ESR accelerates, rheumatic antibodies are positive | DMARD |
| Synovitis | With arthralgia, the pain is often more localized around the joint | Pain with mandibular movement | Wide opening, occlusal trauma | Aseptic infection featured by occlusal interference (e.g., deep overbite, overjet, crossbite, open bite, locked bite, edge to edge) | MRI | NSAID, irrigation, physiotherapy |
| Osteoarthrosis | With arthralgia, the pain is often more localized around the joint | Noises during different time phases of the opening and closing movements, decreased range of motion (or flexibility) | Mechanical loading, cartilage degradation | Although TMJ osteoarthritis has been described as a noninflammatory arthritic condition resulting from degenerative changes of the joint, multiple inflammatory cytokines could have an important role in TMJ osteoarthritis pathogenesis | CT, MRI, arthroscopy | Conservative treatment (restricting jaw movements, analgesics, splint therapy and physiotherapy), intra-articular injections/arthrocentesis (hyaluronic acid, corticosteroids, and platelet-rich plasma), arthroscopy, open joint surgery (e.g., discectomy, condylectomy) |
| Ankylosis of TMJ | TMJ becomes fused by bony or fibrous tissue | Fibrous and/or bony ankyloses limit the opening of the mouth, severely ruining jaw function as well as causing oral hygiene and nutritional problems, particularly affecting jaw growth and development when this condition occurs during the growing years | Trauma, infection, sepsis, previous TMJ surgery, congenital deformities, idiopathic factors, systemic arthropathy (e.g., osteoarthritis, systemic lupus erythematosus, psoriatic arthritis, and gout) | Unilateral TMJ ankylosis results in decreased jaw mobility and function, decreased growth on the involved side, facial asymmetry with the mandible shifted toward the ipsilateral side, retruded mandible, decreased vertical height of the maxilla and mandible on the ipsilateral side, usually a class II occlusion and crossbite tendency on the ipsilateral side, transverse cant in the occlusal plane, evidence of bony ankylosis between the condyle and the fossa, and decreased oropharyngeal airway | Clinical examination and imaging studies, such as plain films, orthopantomograms, CT scans, MRI, and three-dimensional reconstruction | Multiple surgical modalities have been proposed to manage TMJ ankylosis, including gap arthroplasty, interpositional arthroplasty, and TJR |
| Coronoid process hypertrophy/hyperplasia or giant coronoid syndrome | An abnormal volumetric increment of the mandibular coronoid process | Progressive limitation of the mouth-opening ability caused by abnormal elongation of the coronoid process formed of histologically normal bone | The etiopathogenesis is still not conclusive; in the literature, different causes are reported | Absence of pain, presence of an anatomic stop resulting from osseous pathology | Panoramic X-ray, cone beam CT, MRI | Surgical approach (coronoidectomy and coronoidotomy) |
| Lateral pterygoid myospasm | Onset of dull pain that is confined to the deep part of the TMJ and its affiliated tissues (e.g., posterosuperior facet of maxillary tuberosity) at rest and with function | Continuous muscular contraction causing local pain, limitation of movement, and increased EMG activity | Dystonia of stomatognathic system | Acute malocclusion in serious LPM spasm cases | EMG, resting-state functional MRI | Physiotherapy, block therapy, local hot compresses, traditional Chinese medicine |

CT, computed tomography; DMARD, disease-modifying antirheumatic drug; EMG, electromyogram; ESR, erythrocyte sedimentation rate; HLA, human leukocyte antigen; LPM, lateral pterygoid muscle; MRI, magnetic resonance imaging; NSAID, nonsteroidal anti-inflammatory drug; TMJ, temporomandibular joint; TJR, total joint replacement.
